# Supplementary material for: Electrical impedance tomography in pulmonary arterial hypertension
Source: PLoS One. 2021 Mar 17;16(3):e0248214. doi: 10.1371/journal.pone.0248214 (PMC7968654; doi:10.1371/journal.pone.0248214)
Supplement: Attachment — Submitted filename: Response to reviewers.docx [file pone.0248214.s002.docx]

**RESPONSE TO THE REVIEWERS**

We would like to thank both reviewers for the careful evaluation of our manuscript and for their considerations and recommendations.

**Reviewer #1**

We thank the reviewer for all the comments on our manuscript.

**Reviewer #2**

Below, our responses for each one of the questions.

**1. The limited number of patients, in particular of the "healthy" cohort. How were the patients were selected? Were they consecutive? Why patients with normal RHC were referred for the invasive procedure? Were they ill?**

Patients with indication of RHC for PH diagnosis or re-evaluation were consecutively recruited for the study. Those patients referred to RHC for PH diagnosis that presented normal pulmonary arterial pressure levels composed our non-PH population. In a study from our group, about 20% of the individuals that undergo RHC as part of the diagnostic evaluation for the presence of PH presented normal pressure levels (Gavilanes, J Bras Pneumol. Nov-Dec 2014;40(6):609-16). Although limited, this subgroup of patients was not the core of our study that aimed to investigate the potential of EIT in the prognostic assessment of PH patients.

***Modifications to the text:** page 8, lines 205-207.

**2. How PH was suspected? Was it based on transthoracic echocardiography findings?**

For those patients under diagnostic evaluation, PH was suspected by the combination of suggested symptoms/signs plus the finding of systolic PAP ≥40 mmHg in the transthoracic echocardiography. All other PH patients included in the study already had the diagnosis (table 1).

***Modifications to the text:** page 3, lines 89-92.

**3. The authors excluded pts with post-cap PH. I'd suggest to compare pre-cap with post-cap since the most intriguing aspect might be the differentiation between vascular abnormalities in pre and post-capillary PH.**

We thank the reviewer for raising this point. Definitely the comparison of EIT findings in pre and post-capillary PH might help us to better understand the behavior of the pulmonary vascular system in both conditions. Although this particular point is of our utmost interest, it was out of the scope of this work, which aimed to first evaluate the clinical utility of EIT in pre-capillary PH. Nevertheless, the reviewer’s suggestion is within the pipeline of studies addressing the use of EIT in other forms of PH.

**4. Information on feasibility, costs and reproducibility of the test should be provided.**

The reproducibility of EIT has been studied in both ventilation and perfusion studies (Elke G, [Physiological Measurement](https://www.researchgate.net/journal/0967-3334_Physiological_Measurement) 2007, 28(7):S261-7; Reifferscheid F, Respirology 2011, 16(3):523-31; Proença, IEEE Biomedical Circuits and Systems Conference (BioCAS) Proceedings. IEEE, 2014; Braun F, Journal of Clinical Monitoring and Computing (2019): 1-9; Braun F, PLoS One 2018, 16(3): e0191870). In a model for estimating cardiac stroke volume (SV) by means of EIT, compared to cardiac magnetic resonance, a mean error of -10±12.8 mL was found (Proença, IEEE Biomedical Circuits and Systems Conference (BioCAS) Proceedings. IEEE, 2014). Two of these studies showed that the core difficulty in EIT-based SV monitoring is that purely amplitude-based features (that is the case of our study) are strongly influenced by other factors: posture, electrode contact impedance and lung or heart conductivity (Braun F, Journal of Clinical Monitoring and Computing (2020); Braun F, PLoS One 2018, 16(3): e0191870).

The technology consists in installing two auto-adhesive belts over the thorax of a patient by a trained technician. Each belt is connected to a cable that distributes information between the belts and the (trans-)portable EIT device, whose digital/touchable interface also allows a simple operation by a trained technician. These characteristics demonstrate the feasibility of EIT as the growing use of it for bedside evaluation in ICU patients (Bachmann M, Crit Care 22, 263 2018).

It is difficult to discuss the costs of the technology, given the regional characteristics of pricing. Nevertheless, as a basis for comparison the costs of the equipment in our region are somewhat equivalent to an echocardiogram machine.

***Modifications to the text:** page 11, lines 258-67.

**5. Echocardiography represents the screening exam for the identification of PH. I'd appreciate a comparison between the performance of echo and EIT. The superiority/complementarity of the two techniques should be discussed.**

We thank the reviewer for this point. Indeed, the next step in the evaluation of EIT for PAH patients is to analyze its potential as a diagnostic tool. The design of our current study prevents such analysis. A study enrolling only patients at diagnostic evaluation for PH would be necessary for this. The scope of our study was to evaluate the prognostic information that could be derived from EIT measurements, thus focusing mainly in patients with established PH.

Since the performance of EIT was compared with the hemodynamic parameters obtained by RHC, a discussion about superiority/complementarity of EIT and echocardiography would be speculative. Nevertheless, we included in the discussion section the comment on the need for future studies addressing the diagnostic role of EIT in comparison to Echocardiography.

***Modifications to the text:** page 11, lines 268-72.

**6. EIT much strongly correlates with stroke volume. In PAH cohort stroke volume is obviously reduced compared to healthy subjects. How can the authors exclude that EIT could present similar correlation in other diseases determining reduced stroke volume (i.e. HFrEF, valve diseases, etc).**

We agree with the observation: we cannot exclude that EIT could present similar correlation in other diseases determining reduced SV. Unfortunately, as these patients were excluded during selection and we only focused in PAH patients, our findings cannot be considered for conditions such HFrEF or valve diseases. That is certainly a question worth investigating in a dedicated study.

**7. Since most of patients were treatment naïve it’d be interesting to assess the trends of EIT longitudinally after treatment initiation.**

We thank the reviewer for this comment. It is indeed possible that response to treatment initiation bring additional prognostic information. Unfortunately, as per our study design, this analysis was not performed regularly after treatment initiation nor was it performed pre-post treatment initiation. We added this comment to the limitations of our study.

**8. For all the limitations above, the authors should consider to completely eliminate the paragraph on outcome. I don't feel it necessary since the goal of the study is mostly focused on diagnosis rather than prognostication.**

We apologize if the manuscript implied an understanding that our goal was mostly focused in diagnosis. As a matter of fact, our main objective was to explore the EIT as a noninvasive prognostic tool that could be used in an outpatient scenario, the reason why we assessed its ability to reflect disease severity according to RHC findings. We better clarified within the text the main objective of our study.

***Modifications to the text:** page 3, lines 79-82.
