# Supplementary material for: Electrical impedance tomography in pulmonary arterial hypertension
Source: PLoS One. 2021 Mar 17;16(3):e0248214. doi: 10.1371/journal.pone.0248214 (PMC7968654; doi:10.1371/journal.pone.0248214)
Supplement: Attachment — Submitted filename: Response to reviewer.docx [file pone.0248214.s003.docx]

**RESPONSE TO THE REVIEWER**

**Reviewer #3**

We thank the reviewer for all the comments on our manuscript.

Below, our responses for each one of the questions.

**1. Be careful with abbreviations, the use of PAH and PH confuses the reader and it must be ensured that the same abbreviation format is used throughout the text (page 3 line 96).**

We thank the reviewer for this point. We chose to eliminate the abbreviation “PH” referred to pulmonary hypertension and we maintained exclusively the abbreviation “PAH” in reference to pulmonary arterial hypertension.

***Modifications to the text:** page 3: lines 109, 110, 112, 118; page 8: lines 228, 229, 230, 232; page 11: line 302.

**2. A paragraph related to patient follow-up would be appreciated in the methodology section.**

Paragraph included.

***Modifications to the text:** page 4, lines 119-120.

**3. The importance and need of using EIT is not clear, it is a new tool that requires trained personnel and offline analysis with special software, it is expensive (the disposable belts from Timpel) and in the text you mention that "SV provides significant prognostic information " why is this not enough? I suggest to emphasize the importance of multidimensional evaluation in PAH, to justify the use of this new tool.**

We agree with the reviewer that the multidimensional assessment is pivotal in PAH. We better stated the potential role of EIT as a non-invasive tool that provides additional prognostic information as a reflection of the SV behavior.

***Modifications to the text:** page 10, lines 270-274.

**4. Considering the limitations mentioned in the manuscript, I personally find your conclusion quite ambitious. Much remains to be known and validated before saying "could be incorporated in the routine evaluation of PAH patients".
Perhaps you could incorporate a paragraph mentioning future challenges and next steps related to the use of EIT for the diagnosis and follow-up of PAH.**

We thank the reviewer for raising this point.

***Modifications to the text:** page 12, lines 309-312.
